# A systematic review on removal of osseointegrated implants: un update

**DOI:** 10.1186/s12903-023-03438-5

**Published:** 2023-10-13

**Authors:** Giuseppe Tafuri, Manlio Santilli, Eugenio Manciocchi, Imena Rexhepi, Gianmaria D’Addazio, Sergio Caputi, Bruna Sinjari

**Affiliations:** 1 Unit of Prosthodontics, Department of Innovative Technologies in Medicine and Dentistry, University “G. d’Annunzio” of Chieti- Pescara, 66100 Chieti, Italy; 2 Electron Microscopy Laboratory, University “G. d’Annunzio” of Chieti-Pescara, 66100 Chieti, Italy

**Keywords:** Implants, Dental implant, Explantation, Removal, Systematic review

## Abstract

**Background:**

Today dental implants represent an effective therapy in case of partial or total edentulism, with an excellent success rate. Despite the results obtained, there may be biological or mechanical complications during the therapy, which lead to the loss of the implant. This systematic review aims to evaluate the current state of the art in the literature on techniques used for the removal of dental implants. Various aspects will be analyzed, such as the success of the technique, any complications, and the advantages and disadvantages of their use.

**Methods:**

Two reviewers conducted a literature analysis (PubMed, Embase, Web of Science) of the last 20 years (2003–2023). The main criterion analyzed was the success of the technique, while secondary outcomes such as complications and risks of the technique were also analyzed. 258 articles were identified in the various search databases. 42 eligible articles were subsequently identified after an article screening. Only 18 full texts were subsequently included in the review.

**Results:**

A total of 18 articles were selected and 1142 implants and 595 patients were included. The main techniques used were the Counter-Torque Ratchet Technique (CTRT), Piezoelectric bone surgery (PBS), trephine drills, carbide burs, Erbium, Chromium, Yttrium, Scandium, Gallium, Garnett (Er:Cr:YSGG) laser and carbon dioxide (CO_2_) laser. Combined uses of techniques have been identified such as: PBS and trephine burs or carbide burs, trephine burs with the use of a 3d-printed guide, CTRT and trephine burs. The technique with the highest success rate, less morbidity for the patient, and less removal of bone appears to be the CTRT.

**Conclusions:**

The use of conservative techniques, especially CTRT, in bone removal is useful to allow for immediate implant placement in the removal area. However, further studies with a high sample size are needed to be performed on all techniques, particularly new randomized controlled trials (RCTs) that allow for the analysis of the success of alternative techniques such as Laser and Piezosurgery, which appear to be very promising.

## Background

Today, dental implants represent an effective therapy for rehabilitating patients with complete or partial edentulism. The long-term results and prospects are excellent, with a success rate of around 90%, with better results in implant therapies performed on the mandible [1, 2]. Single-tooth implant therapies have been found to be more effective than those in edentulous or overdenture cases [3]. However, despite long-term successes, the oral health-related quality of life of patients rehabilitated with implant-supported dental prostheses has not demonstrated an overall superiority over conventional prosthetics [4]. There may be complications that lead to failure, classified by Esposito et al. as biological, mechanical, iatrogenic, and inadequate implant adaptation [5]; these complications can lead to treatment failure with implant loss [5]. Implants at the tissue level, inserted following thorough periodontal treatment and regular supportive periodontal care, lead to positive long-term outcomes. Nevertheless, individuals who have a prior history of periodontal disease and do not adhere to supportive periodontal care are at a greater risk of biological complications and may face a greater risk of implant failure [6]. Implant failure has been also classified as early or late. In early failure, no osseointegration has occurred, and the implant is lost early, while in late failure, there is initial osseointegration that is lost over time due to biological or mechanical reasons [7]. The most common complication is peri-implantitis [2, 8]. Peri-implantitis has been defined by the European Federation of Periodontology (EFP) as a pathological condition where there is inflammation of the peri-implant mucosa and loss of supporting bone [9]. The main risk factors for peri-implantitis have been identified primarily as smoking [10, 11] and a previous history of periodontitis [12]. It has been reported in the literature that excess cement associated with poor oral hygiene can also promote the onset of peri-implantitis [13]. Additional scientifically substantiated factors that may have an impact consist of inadequate patient selection, insufficient periodontal treatment, absence of diagnosis and control of peri-implant mucositis, and irregular peri-implant/periodontal support therapy [14]. Peri-implant mucositis is an inflammatory condition in the soft tissues surrounding an osseointegrated implant when there is no loss of supporting bone or ongoing marginal bone deterioration [15]; it causes a greater infiltration of inflammatory connective tissue and a higher frequency of bleeding around implants compared to teeth. The tissue loss at experimental peri-implantitis sites is more pronounced than that observed at experimental periodontitis sites [16]. Although periodontitis and peri-implantitis lesions share similarities in their origin and exhibit similar clinical features, from a pathophysiological perspective, they represent distinct entities [14]. Systemic factors such as immunosuppression, uncontrolled chronic diseases such as diabetes, favor the onset of the disease [17]. The type of bone can also influence implant success, as Chrcanovic et al. have reported that implants inserted into type IV bone are more prone to failure [18]. There are also patient-related factors such as the presence of parafunctional habits, such as bruxism, which can be risk factors for implant survival [19]. Another determining mechanical factor in fracture is the position of the implant in the oral cavity, the implant material, and its prosthetic components. At the prosthetic level, the outcome is influenced by the implant-abutment connection used [20]. In case of failure, implant removal must be performed. There are several methods for implant removal. The first parameter to consider is to evaluate whether the implant is osseointegrated or not; if there is no osseointegration, its removal will be simpler; conversely, where there has been osseointegration, removal will be more invasive and difficult. Another parameter to consider is the future planning of another implant in the removal site; if planned, too invasive removal methods should be avoided, which would cause excessive loss of bone volume [21].

## Methods

The following systematic review was conducted in accordance with the Preferred Reporting Items for Systematic review and Meta-Analysis Protocol (PRISMA-P) and Needleman's recommendations [22, 23] (Table 1).

**Table 1:** Search strategy according to the focused question (PICO)

Focused Question (PICO)	What are the most used techniques in implant removal?	
**Search strategy**	**Population**	Osseointegrated dental implants to be removed
	**Intervention**	Removal of dental implants
	**Comparison**	Removal by non-conservative methods
	**Outcome**	Success and clinical application of these techniques

### Focused question

The question was defined in accordance with the Population, Intervention, Comparison, Outcome method (PICO) [24]. The review focuses on subjects requiring dental implant removal. Different implant removal techniques will be compared.

### Primary outcome

The success of individual implant removal techniques will be evaluated. Success rate refers to the implants that were successfully removed using a single technique.

### Secondary outcome

The amount of residual bone after implant removal and the presence of any complications will be evaluated. Based on this parameter, it will be possible to determine which technique is more conservative.

### Inclusion criteria

Publications were included in the study based on the following inclusion criteria: human studies, osseointegrated dental implants, detailed description of the technique used for removal, and reasons for implant removal. The implants considered are dental implants.

### Exclusion criteria

The study excludes reviews, systematic reviews, meta-analyses, in vitro studies, animal studies, expert opinions, communications, studies without full text, removal of zygomatic, pterygoid, basal, and bicortical implants.

### Search strategy

A systematic review of the literature was conducted using online electronic databases (Pubmed, Embase, Web of Science). The last 20 years of publications (2003–2023) were considered. The electronic search was conducted using the following terms: "dental implants" OR "implant" OR "oral implant" AND "explantation", "dental implants" OR "implant" OR "oral implant" AND "removal", "dental implants" OR "implant" OR "oral implant" AND "failure", "dental implants" OR "implant" OR "oral implant" AND "complications"; "failing implant" AND "implant removal".

### Study selection

Two independent reviewers (G.T, M.S,) reviewed the titles and abstracts of each work. Incongruent results were discussed; Cohen's kappa was used to evaluate inter-reviewer agreement.

### Data extraction

Various parameters were analyzed in the studies, such as study design, number of patients, number of implants removed, removal technique, success of removal technique and immediate implant placement.

### Ethical considerations

No personal information of patients was used in this systematic review. All data was extracted from publicly available scientific publications. No informed consent or ethical approval was required for this systematic review.

## Results

### Literature search

**Fig. 1:**
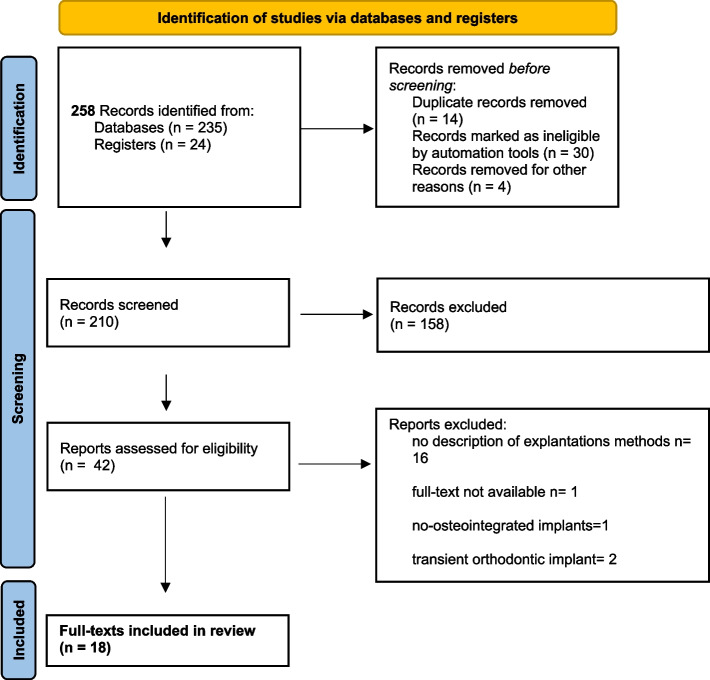
Prisma flowchart for included studies

The authors conducted a literature search and identified 258 articles through an electronic database search. After screening and removing duplicates, 210 titles were considered for further consideration. Of these, 42 articles were reviewed in detail, and 18 publications were ultimately included in the analysis. The quality of the included studies was assessed, and all publications were at a high risk of bias, except two studies that have a largest sample size. The authors excluded 24 studies from the final analysis due to various reasons, such as unclear descriptions of explantation techniques, and studies exclusively describing explantation of non-osseointegrated implants, orthodontic transient implants and full-text non available (Fig. 1).

### Exclusion criteria

Two hundred fifty-eight studies that were carefully reviewed were excluded from the final analysis (Fig. 1). The main reasons for exclusion were:

1. The study being a review or expert opinion,
2. An unclear description of the explantation technique used,
3. The study exclusively described explantation of non-osseointegrated, blade, palatal implants,
4. Implants were retrieved solely for histomorphometric purposes, and [5] full-text articles could not be obtained.

### Literature analysis

The following literature review led to the selection of 18 articles with a total of 1141 implants and 594 patients. The types of studies reported are case reports [13], case series [1], retrospective studies [4]. The techniques described are mainly 4: CTRT, PBS, Laser explantation and Trephine burs. The most conservative method was found to be CTRT, which due to the greater preservation of residual bone also allowed the reinsertion of implants (Table 2). In the study by Anitua & Orive, 35 implants out of 91 (38.5%) were reimplanted after removal of the implant. This technique was mainly used in biological complications such as peri-implantitis in 82.9% of cases (131 implants) in the study by Anitua et al. 2016, 86.2% of the cases Anitua et al. 2020 and Stajic et al. 2016. In two of these studies, this technique was complemented by the use of a trephine drill to remove the initial few millimeters of bone. The other causes of removal were identified as fracture [10] and malpositioned implants [3]. In only four studies, the sample size was determined to be sufficient; the remaining studies carry a high risk of bias due to their small sample sizes. Other methods for implant removal have solely been documented in case reports. The primary alternative techniques include the use of a trephine bur, high-speed burs, laser explantation, and piezosurgery. These methods have been employed for the extraction of fractured fixtures or abutments. Deeb et al. [20] introduced a modification to the trephine bur technique, which involves a 3D-printed guide in conjunction with trephine burs. An unconventional application of the CO2 laser was proposed by Worni et al. [21, 25, 26]; in this study, thermo-necrosis is induced at the level of the implant connection. In the following week the implant can be removed with a low torque (37 Ncm). However, there is only one case of extraction of an implant reported with this method. An article combines the use of trephine bur with forceps and elevator, while in another one [27] combines the use of trephine and burs with piezosurgery. The exclusive use of trephine drills is reported in cases of fixture and/or abutment fracture. Two studies combine the use of trephine burs with GBR for new implant placement [28, 29]; an article instead adds a sinus floor elevation to this method [30]. One study used high-speed burs [31]: diamond and carbide burs (round and fissure) with irrigation combined with the use of forceps and elevators. However, the bone removal of the fractured abutment with these methods was excessive. Subsequently, a larger diameter implant had to be inserted.

**Table 2:** Results of literature search

Authors	Type of study	N° patients	N° implants	Implant brand	Technique of explantation	Reason of explantation	Success
(L.P. & T., 201﻿﻿0) [25]	Case report	1	1	Nobel Biocare ®	(Er,Cr:YSGG) Laser	Perimplantitis	Yes, this procedure provides an effective option to conventional removal techniques
(Jin et al., 2017) [21]	Case report	4	4	ND	Trephine + GBR	Fracture	Yes, the author inserted a new implant fixture in the same site
(Anitua & Orive, 2012) [26]	Retrospective longitudinal study	42	91	3i®, Nobel Biocare ®, Importacion Dental, Pitt-Easy®, TRS, Astra®, Defcon®, Osteoplus®, Straumann®	Counter-torque (BTI extraction kit); combination of the extractors with a new trephine kit (13 implants)	ND	Yes, in 35 of the 91 implants (38.5%), after removing the implants, new implants were placed
(Anitua et al., 2020) [27]	Retrospective study	355	759	ND	Counter-torque	Biological complications (86.2%); mechanical complications (11.9%); surgical intervention (1.9%)	Yes; Successful implant explantation was achieved in 98.4% of the implants. The frequency of complications was 1.3%
(Anitua et al., 2016) [32]	Retrospective study	81	158	ND	Counter-torque (The use of trephine burs to cut into the first 3 to 4 mm was necessary in 19 explantations)	Periimplantitis (131 implants; 82.9%) followed by malpositioning of the implants (22 implants; 13.9%)	Yes, the technique maximally preserved the available bone tissue and minimized the necessity for advanced tissue regeneration procedures
(Messina et al., 2018) [29]	Case report	10	ND	ND	Piezosurgery (Mectron Medical technologies, Carasco, Italy)	Fracture	Yes, primary wound closure without any soft tissue dehiscence and no healing problems during the postoperative period
(Marini et al., 2013) [30]	Case report	1	1	ND	Piezosurgery	Malpositioned implant	Yes, piezosurgery may reduce the risk of postsurgical life-threatening complications
(Oguz et al., 2015) [22]	Case report	1	2	(Astra Tech ®, Mölndal, Sweden) (3.5 × 11 mm and 4 × 13 mm)	Trephine + GBR	Fracture	Yes, the fractured implants were removed, bone grafting was performed, and implants with a larger diameter were placed
(Deeb et al., 2018) [31]	Case report	1	2	ND	3D—printed guide and trephine	Fracture	Yes, Computer-aided planning and trephine burs can be used for precise implant removal
(Muroff, 2003) [23]	Case report	1	1	Nobel Biocare®	Trephine and conservative sinus flap elevation	Fracture	Yes
(Li & Chou, 2014) [24]	Case report	1	1	Ankylos ®	Diamond bur; Carbide bur with irrigation (round and fissure bur); forceps; elevator	Abutment Fracture	Yes, the surrounding bone was not significantly compromised, and a larger implant was not necessary for immediate replacement
(Worni et al., 2018) [33]	Case report	1	1	Straumann® (Bone Level 4 × 10 mm RC implants, Institut, Straumann)	CO_2_ Laser-induced thermo-necrosis (Lutronic Denta 2, Lutronic) Counter torque	Malpositioned implant	Yes, after one week from the laser-induced thermo-necrosis, the implant could be removed at 37 Ncm
(Lee, 2017) [34]	Case report	2	4	ND	Trephine, forceps, elevator (I case); counter torque with Neo FR Kit (NeoBiotech, Seoul, Korea)	Fracture (case I); Periimplantitis (case II)	Yes, a new implant was placed after 6–8 months
(Stajčić et al., 2016) [35]	Retrospective cohort study	81	95	Nobel Biocare®, Straumann®	Trephine, counter-torque with Neo Fixture Remover Kit (Neobiotech Co., Korea), neo bur–elevator–forceps (ηBEF) technique	Fracture; Periimplantitis;	Yes, the CTRT technique appeared to be the most elegant technique with the highest predictability for insertion of another implants
(Cardoso et al., 2010) [32]	Case report	1	2	ND	Flap + Trephine and autogenous bone blocks	Fracture (trauma)	Yes, satisfactory results have been shown as regards soft and hard tissues wound healing
(Covani et al., 2006) [36]	Case series	9	9	ND	Flap + burs	Fracture	Yes, residual bone defects were observed or probed around any implant at the second-stage surgery, and all implants were asymptomatic and stable
(Matsumoto et al., 2018) [37]	Case report	1	1	ND	CTRT (Implant Retrieval Tool; Nobel Biocare)	Malpositioned implant	Yes, good esthetics as well as functional outcomes
(Dvorak et al., 2012) [20]	Case report	1	4	ND	Piezosurgery, burs and trephine	Fracture	Yes, trephine burr osteotomy is a highly invasive method, and issues such as overheating leading to complications in wound healing and collateral damage to adjacent teeth have been observed

### Quality assessment

The quality of these studies was evaluated by Quality Assessment of Diagnostic Accuracy Studies (QUADAS-2) tool, as shown in Table 3. The test is based on the evaluation of 4 domains such as: patient election, index test, reference standard, flow and timing [38]. Among the 18 publications, 1 was a case series [36] and 13 were case reports that described one to nine cases. Only 4 publications reported on relatively large sample sizes [32, 34, 35, 39]. Additionally, none of the studies included control groups or any blinding measures. As a result, all 18 publications were deemed to be at a high risk of bias.

**Table 3:** QUADAS-2 tool quality assessment, ** high risk of BIAS, * low risk of BIAS

Authors	Patient selection	Index test	Reference standard	Flow and timing
L.P & T., 2010 [33]	**	**	**	**
Jin et al., 2017 [28]	**	**	**	**
Anitua & Orive, 2012 [34]	*	**	**	**
Anitua et al., 2020 [35]	*	**	**	**
Anitua et al., 2016 [32]	*	**	**	**
Messina et al. 2018 [40]	**	**	**	**
Marini et al., 2013 [37]	**	**	**	**
Oguz et al., 2015 [29]	**	**	**	**
Dee et al., 2018 [25]	**	**	**	**
Muroff et al., 2003 [30]	**	**	**	**
Li & Chou, 2014 [31]	**	**	**	**
Worni et al., 2018 [26]	**	**	**	**
Lee et al., 2017 [41]	**	**	**	**
Stajcic et al., 2016 [39]	*	**	**	**
Cardoso et al., 2010 [42]	**	**	**	**
Covani et al., 2006 [43]	**	**	**	**
Matsumoto et al., 2018 [44]	**	**	**	**
Dvorak et al., 2012 [27]	**	**	**	**

## Discussion

**Fig. 2:**
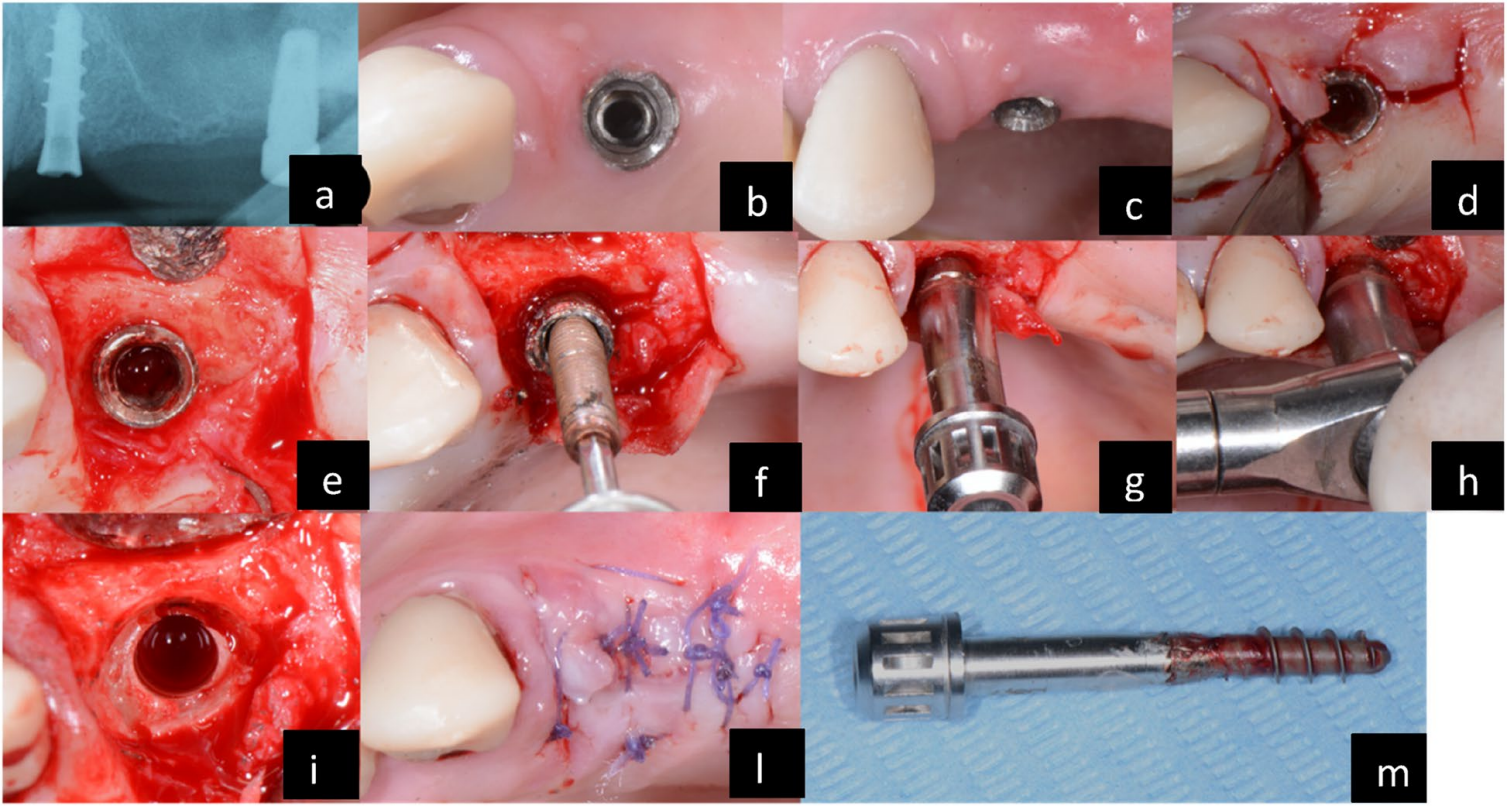
Procedure of a CTRT implant removal. Periapical radiograph and preoperative status (**a**-**c**), mucoperiosteal flap (**d**), implant exposure (**e**), use of counter torque removal tool (**f**–**h**), implant removal (**i**), suturing (**l**), extracted implant (**m**)

This systematic review examined various techniques available for removing osseointegrated oral implants. The 18 studies selected for the review reported successful explantation of all 1141 implants in 594 patients using these techniques. Among the methods, the counter-torque ratchet technique (CTRT) was most frequently used because it seems to be the most conservative technique and will allow the reinsertion of a new implant in the same site or close to it [32, 34, 35, 39] (Fig. 2). This result has also been confirmed by other reviews on the subject, which consider this technique, when feasible, the first choice, despite the trephine drill technique being the better known [21, 39, 45–47]. The counter-torque technique achieved a success rate of 87.7%. Using trephine burs for removal resulted in a success rate of 94%, and the carbide burs technique achieved a 100% success rate. However, these approaches are better suited for the extraction of implants surrounded by a substantial amount of bone. It's advised to perform mucoperiosteal flap elevation for adequate access and visibility in each technique [47]. Implant placement in sites that had previously experienced failure, whether it occurred early or late, leads to a survival rate ranging from 71 to 100% over an average period of 69.4 months [48]. The literature demonstrates that placing a second implant either after implant failure or removal is favorable in terms of future implant survival [48]. A systematic review found that survival rates after a second attempt were approximately 88% over a mean follow-up period of about 40 months [45]. Simultaneous bone regeneration procedures and the use of a removal kit have significantly reduced the impact on dimensional changes, resulting in less discomfort for the patient and a better cost–benefit ratio [49]. Guided bone regeneration is frequently used to reduce the clinical consequences of implant removal. However, implant removal does not appear to impact patient’s satisfactions or their quality of life. Nevertheless, some patients expressed hesitation about undergoing future implant placement at the same clinic or with the same healthcare provider [50]. Trephine burs were recommended for cases with fractured implants or abutment or when torque values exceeded 200 Ncm. In one study, mixed techniques were used with the use of a trephine drill for the first millimeters and counter-torque kit [34]. These techniques are highly invasive for both soft and hard tissues, and the risk of vascular damage is further increased, especially in the case of mandibular implants. Therefore, immediate implant placement in these scenarios is not recommended [48]. The use of mucoperiosteal flap elevation was not always necessary, but explantation kits could be costly due to the need for disposable extraction inserts. Implant design, length, and surface modification also affected removal torque, with acid-etched, particle-blasted, and oxidized surfaces showing higher torque values than those with titanium plasma-sprayed surfaces [32].

The ability to remove the implant with this technique depends on various factors, such as the implant connection, shape, implant geometry, and osseointegration around the implant. It is one of the most effective techniques for implant removal, but certain parameters must be considered. The connection must be in perfect condition, and narrow implants may be prone to fracture. Therefore, this method should be avoided if the connection is not in good condition.

CTRT kits have three components: the first part is screwed into the implant, followed by a driver and then a torque catcher that measures the correct torque for implant removal. The systems are universal and suitable for most implants. There are various counter-torque technique’s kits on the market (Straumann®), the Neo Fixture Remover Kit (Neobiotech®), BTI Implant Extraction System (Biotechnology Institute S.L. ®) Implant Retrieval Tool (Nobel Biocare ®). The suitable extraction screw is selected based on the pitch of the implant connection's threading. The screw is disposable, and its threads must be inserted into the implant connection. It is tightened with a maximum torque of 60 Ncm. The driver for the implant extractor is selected based on the implant's platform. The implant extraction screwdriver has an internal thread that screws into the extraction screw. On average, a torque of 300 Ncm is used for implant removal. The removal movement must be done slowly, and it can cause discomfort to the patient because the forces are high. It is recommended to maintain the patient's jaw during the procedure. After passing the osseointegration stage, the implant is manually removed. This is one of the most conservative ways to remove an implant because the surrounding bone is preserved for the future placement of another implant in the site [34, 35].

The piezo bone-surgery (PBS) can be used to remove supporting bone around the implant to facilitate its removal. The advantage of the piezoelectric device is that it cuts flat bone well, avoiding damage to soft tissues. It has also been shown to improve postoperative bone healing compared to conventional high-speed drills. When performing deeper bone incisions, the piezoelectric device tends to become less efficient, and it is advisable to pause periodically to prevent the tip from overheating as the cutting speed decreases. This instrument works at a frequency that oscillates between 24,000 and 29,500 Hz, which is ideal for preserving sensitive structures [37, 40]. An osteotomy is performed with diamond inserts in the bone-implant interface. the bone is cooled using a saline solution. The osteotomy is performed as close to the implant surface to remove only the minimum necessary amount of bone. This method of removal has mainly been used on fractured implants or abutments improved healing was observed compared to trephine bur surgery [51]. Caution should be exercised in patients with pacemakers, although an in vitro study showed no side effects, further studies are still needed [52].

High-speed drills are used for bone tissue removal, but there may be contraindications, such as the invasiveness of the procedure and the formation of possible emphysema caused by the high-pressure air turbine's exit. Other complications may include stress fractures of the mandible and osteomyelitis. A small-sized drill should be used with constant irrigation to prevent implant parts from dispersing into the oral cavity [21].

The removal technique using a Trephine drill is a common approach but it’s more invasive than CTRT and should only be used when necessary. Drills of various sizes can be used, and the smallest drill must be used, with the diameter slightly larger. The preferred cutting speed ranges from 1200 to 1500 rpm. When half of the bone is removed, elevators, pliers, and dynamic keys can be used to remove the implant [51]. An interesting procedure to simplify implant removal was illustrated by Deeb et al. through which computer planning and a custom fabricated 3D-printed surgical guide were used to assist in the removal of the implant. However, the study does not have a sufficient sample size to validate the technique [25].

**Fig. 3:**
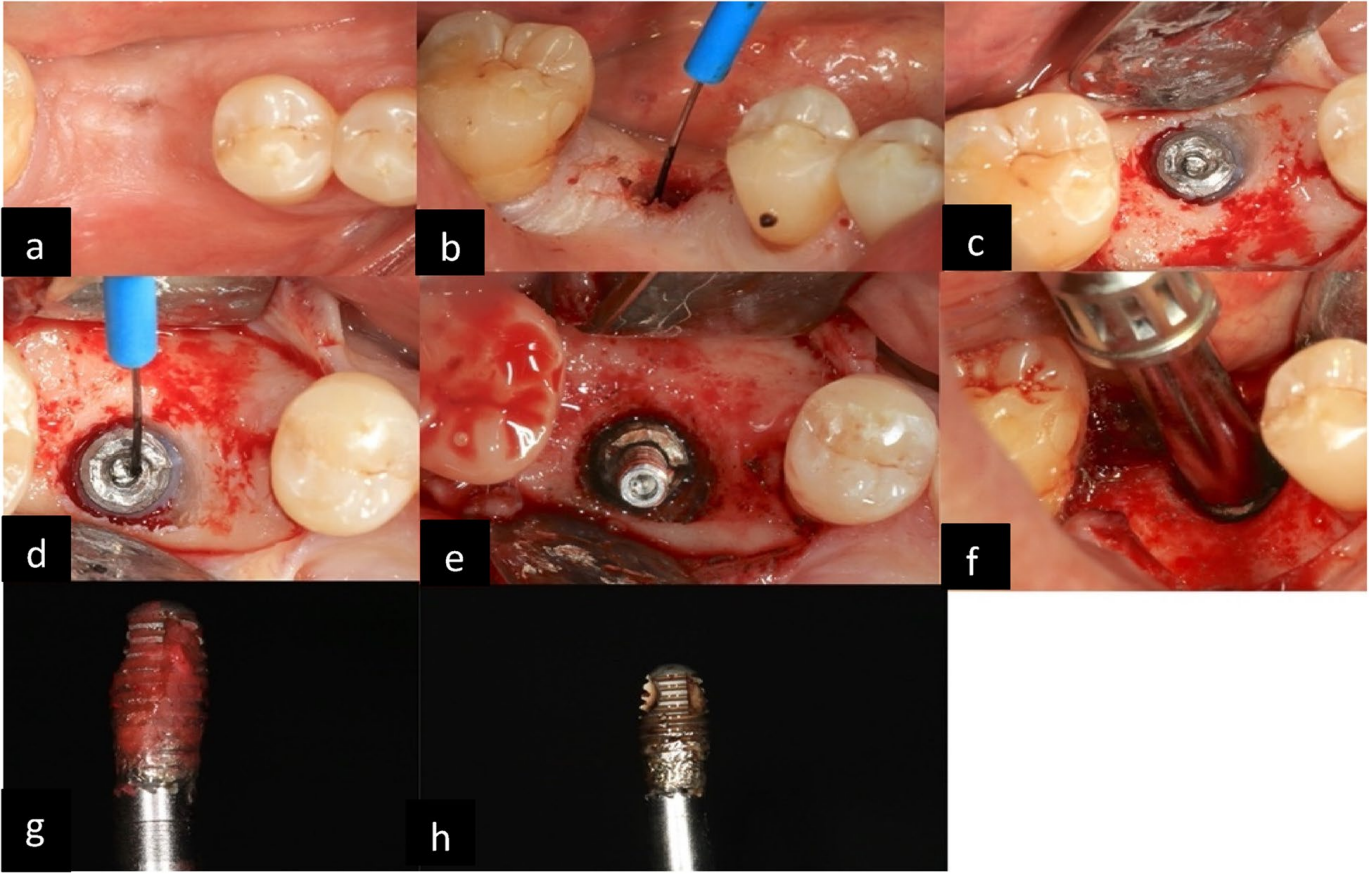
Combination of two procedures; Thermo-explantation and CTRT. Preoperative status (**a**), use of electrosurgery unit to remove mucosal tissue (**b**), implant exposure (**c**), use of electrosurgery unit to place in situ counter-torque removal tool (**d**-**f**), implant removal (**g**), decontamined surface (**h**)

Laser technology is widely used in dentistry, including implant removal. It is non-invasive and allows precise cutting of soft and hard tissues. Laser energy is absorbed by the water and minerals in the tissues, leading to tissue ablation, cutting, and coagulation. Lasers can reduce postoperative pain and swelling, but they are more time-consuming and require specialized equipment. It allows excellent visualization of the operating field with excellent hemostasis. The laser procedure involves a selective removal of the peri-implant bone, as highlighted by Smith and Rose this method is less invasive than the other techniques [53] (Fig. 3). Other in vitro studies have confirmed the less invasiveness compared to other techniques, especially the trephine burs [54].

## Conclusion

Conservative techniques such as CTRT are recommended, which also allow the insertion of a new implant in the same session. If reverse torque is not successful, resective techniques may be used in a careful and conservative manner. When removing an osseointegrated oral implant, factors such as the implant's design, proximity to important structures, and the feasibility and timing of future implant placement should be considered. Further studies with a high sample size are needed to be performed on all techniques, particularly new Randomized Controlled Trials (RCTs) that allow for the analysis of the success of alternative techniques such as Laser and Piezosurgery, which appear to be very promising in bone preservation.

## Data Availability

All data are included into the manuscript.

## Abbreviations

CTRT: Counter-torque ratchet technique
Er: Cr: YSGG: Erbium, Chromium, Yttrium, Scandium, Gallium, Garnett
C0_2_: Carbon dioxide
EFP: European Federation of Periodontology
PBS: Piezoelectric bone surgery
GBR: Guided bone regeneration
RPM: Revolutions per minute
RCTs: Randomized controlled trials
PICO: Population, Intervention, Comparison, Outcome
PRISMA: Preferred Reporting Items for Systematic Review and Meta -Analysis Protocol
QUADAS: Quality Assessment of Diagnostic Accuracy Studies
